# Immunomodulatory mechanisms of lactobacilli

**DOI:** 10.1186/1475-2859-10-S1-S17

**Published:** 2011-08-30

**Authors:** Jerry M Wells

**Affiliations:** 1 Host-Microbe-Interactomics, University of Wageningen, Animal Sciences Department, P.O. Box 338, 6700 AH, Wageningen, The Netherlands

## Abstract

**Abstract:**

Over the past decade it has become clear that lactobacilli and other probiotic and commensal organisms can interact with mucosal immune cells or epithelial cells lining the mucosa to modulate specific functions of the mucosal immune system. The most well understood signalling mechanisms involve the innate pattern recognition receptors such as Toll-like receptors, nucleotide oligomerization domain-like receptors and C-type lectin receptors. Binding of microbe-associated molecular patterns with these receptors can activate antigen presenting cells and modulate their function through the expression of surface receptors, secreted cytokines and chemokines. *In vitro* the cytokine response of human peripheral blood mononuclear cells and dendritic cells to lactobacilli can be strikingly different depending on both the bacterial species and the strain. Several factors have been identified in lactobacilli that influence the immune response *in vitro* and *in vivo* including cell surface carbohydrates, enzymes modifying the structure of lipoteichoic acids and metabolites. In mice mechanistic studies point to a role for the homeostatic control of inducible T regulatory cells in the mucosal tissues as one possible immunomodulatory mechanism. Increasing evidence also suggests that induction of epithelial signalling by intestinal lactobacilli can modulate barrier functions, defensin production and regulate inflammatory signalling. Other probiotic mechanisms include modulation of the T cell effector subsets, enhancement of humoral immunity and interactions with the epithelial-associated dendritic cells and macrophages. A major challenge for the future will be to gain more knowledge about the interactions occurring between lactobacilli and the host *in vivo* and to understand the molecular basis of innate signalling in response to whole bacteria which trigger multiple signalling pathways.

## Introduction

The genus *Lactobacillus* comprises a large heterogeneous group of low-G+C Gram-positive, non-sporulating, and facultative anaerobes [1]. Taxonomically, the genus *Lactobacillus* belongs to the phylum Firmicutes, class Bacilli, order *Lactobacillales*, family *Lactobacillaceae*. Currently the genus *Lactobacillus* contains 154 species of which more than 20 have been sequenced. They have limited biosynthetic abilities, and require preformed amino acids, B vitamins, purines, pyrimidines and (usually) a sugar as a carbon and energy source which is fermented to produce lactic acid as a common end product. These nutritional requirements restrict their habitats to those in which the required compounds are abundant. Nevertheless, the lactobacilli occupy a variety of niches including milk, plant surfaces, and the gastrointestinal tract of humans and other animals. Several food-associated species of *lactobacilli* have an excellent safety profile and a “generally-regarded-as-safe” status in the food industry due to their long history in food fermentation and human consumption.

Lactobacilli are found in low numbers in the small intestine of adults but some may originate from fermented foods or the oral cavity which is home to several autochthonous species. In adult faeces they form only a minor component of the microbiota ranging from 0.01 to 0.6% of total counts [2]. In infants lactobacilli are present in the faeces in variable amounts ranging from 10^5^ to 10^8^ CFU/g with *L. salivarius*, *L. rhamnosus*, and *L. paracasei* being common species [3]. In addition lactobacilli are dominant members of the human vaginal microbiota where they play a protective role against urogenital infections [4].

Numerous probiotic studies with different strains of *Lactobacillus* have been performed in humans and murine models to investigate their probiotic potential. While some clinical studies have been negative or inconclusive [5-8] others have shown positive results (see e.g. [9-14]). The lactobacilli have given significant and promising results in treating acute infectious diarrhoea and in the prevention of antibiotic-associated diarrhoea in human clinical trials [10]. The use of probiotic lactobacilli in the treatment and prevention of allergic diseases and in the treatment of allergic rhinitis/asthma has been reviewed recently [15,16]. Most clinical studies on allergy have been performed with *Lactobacillus rhamnosus* GG (LGG) which was shown to prevent atopic eczema or dermatitis [13,14]. Subsequent studies on the use of LGG in the treatment of atopic eczema suggested a therapeutic effect [9,11,12], whereas more recent studies show no therapeutic or preventative effects benefits in the development of sensitization and atopic disease, particularly in infants with atopic dermatitis [6-8]. These contrasting results probably reflect the inherent complexity of the allergic syndrome, and differences in the clinical set-up e.g. different target populations, countries, intervention schemes and the formulation of LGG used in the study [15]. Overall there is encouraging evidence that specific lactobacilli probiotics are valuable in the prevention and treatment of different diseases but their successful application would benefit greatly from a better understanding of the mechanisms of probiotic action in clinical studies. The purpose of this review is to discuss the molecular basis for immune recognition of lactobacilli by the host and to highlight the possible mechanisms leading to different strain-dependent host responses. A detailed analysis of results of probiotic studies in different mouse models and clinical studies is beyond the scope of this review but the immunomodulatory mechanisms of probiotic lactobacilli *in vivo* are discussed.

### Immune recognition of lactobacilli

Lactobacilli can elicit innate and adaptive immune responses in the host via binding to pattern recognition receptors (PRR) expressed on immune cells and many other tissues including the intestinal epithelium. PRR recognize conserved molecular structures known as microbe-associated molecular patterns (MAMPs) and signal to induce the production of cytokines, chemokines and other innate effectors (recently reviewed [17-19]). These signalling receptors can be divided into three families; Toll-like receptors (TLRs), retinoic acid inducible gene I (RIG-I)-like receptors that recognize viral RNAs (RLRs) and nucleotide oligomerization domain-like (NOD) receptors (NLRs). The TLR family is the best characterized and 10 TLRs have been identified in humans and 12 in mice [19]. For TLRs it has been shown that MAMP binding and specificity is achieved through the arrangement and sequence variation in the extracellular leucine-rich repeat (LRR) domains. Dimerization of the TLR and the highly conserved intracellular Toll-interleukin 1 receptor (TIR) domains leads to the recruitment of adaptor molecules such as myeloid differentiation primary response gene 88 (MyD88), toll-interleukin 1 receptor (TIR) domain containing adaptor protein (TIRAP) and TIR-domain-containing adapter-inducing interferon-β (TRIF) to initiate the signalling cascade. Each PRR recognizes a specific molecular pattern and can be expressed on the cell surface, in intracellular compartments or in the cytosol (Table 1). TLR1, 2, 4, 5, 6 and 11 recognize mainly microbial membrane components and are expressed on the cell surface, TLR3, 7, 8 and 9 recognize nucleic acids of bacterial and viral origin and are expressed in intracellular compartments such as the endoplasmic reticulum, endosomes, lysosomes and endolysosome (Table 1).  In epithelial cells TLR9 is expressed on the cell surface and intracellularly.

**Table 1:** PRR, ligands and subcellular localization

Receptor	Localization	Ligand	Origin of the ligand
TLR2	Cell surface	Lipopeptides	Bacteria
		Lipoproteins	G+ bacteria
		LTA	G+ bacteria
TLR2/1	Cell surface	Triacylated lipopeptide	G-bacteria, mycoplasma
TLR2/6	Cell surface	Diacylated lipopeptides	G+ bacteria, mycoplasma
TLR3	Intracellular compartment	dsRNA	Viruses, virus infected cells
TLR4/MD2	Cell surface, intracellular compartment	LPS	G- bacteria
TLR5	Cell surface	Flagellin protein	Bacteria
TLR7	Intracellular compartment	ssRNA	Viruses
TLR8	Intracellular compartment	ssRNA	Viruses
TLR9	Intracellular compartment, cell surface	DNA	DNA viruses, bacteria
TLR11	Cell surface	Uropathogenic bacterial components	Uropathogenic bacteria
NOD1	Cell cytoplasm	Meso-DAP	PG from G-, some G+, mycobacterium
NOD2	Cell cytoplasm	MDP	PG from G-, G+ bacteria, mycobacterium

PG, peptidoglycan; LTA, lipoteichoic acid; LPS, lipopolysaccharide; DAP, diaminopimelic acid; MDP, muramyl dipeptide; G+, Gram positive; G-, Gram negative; ssRNA, single-stranded RNA; dsRNA, double-stranded RNA.

**Figure 1:**
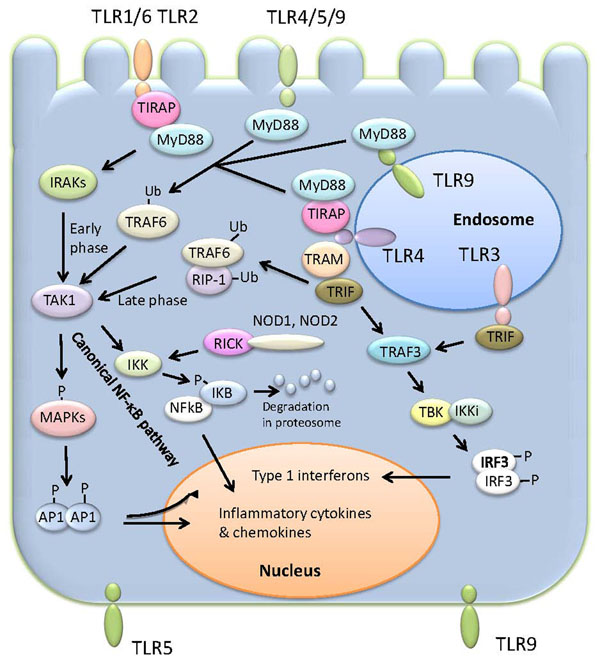
****Simplified scheme for TLR signaling in enterocytes.**** TLRs in the cytoplasmic membrane or subcellular compartments such as the endosome bind the MyD88 adapter protein to initiate signaling. MyD88 recruits TRAF6 and members of the IRAK family (IRAKs) which leads to activation of the TAK1 complex (TAK1, TAB1/2). The activated TAK complex then activates the IKK complex (IKKα and IKKβ) which phosphorylates the inhibitor of NF-κB (IKB) leading to its degradation and the translocation of NF-κB (typically p50 and p65 heterodimers) into the nucleus where it activates gene expression. The activated TAK1 complex simultaneously activates the MAPK pathway resulting in phosphorylation (P) and activation of the transcription factor AP1. The canonical pathway of NF-κB activation and can also be triggered by binding of TRAM and TRIF adaptor proteins to TLR4 (not shown). The adaptor protein TRIF which binds to TLR3 recruits TRAF3 which interacts with the TBK and IKKi kinases to promote phosphorylation (P) of IRF3 which translocates to the nucleus and activates transcription of type 1 interferons, especially IFN-β. The NOD1 and 2 receptros activate NK-κB via the serine threonine kinase RICK. This diagram is a modified version of Figure 1 published by Wells et al., 2010 [17].

TLR signalling pathways have been reviewed in detail recently [19] and except for TLR3 involve recruitment of MyD88, which in turn activates the mitogen-activated protein kinase (MAPK) pathway and the nuclear factor κB (NF-κB) pathway signalling cascades [20] (Fig 1). TLR3 utilizes the adaptor protein TRIF leading to the expression of type 1 interferons. In addition this adaptor serves as an alternative adaptor to MyD88 in the TLR4 signalling pathway. The NOD receptors activate the MAPK pathway and the canonical NF-κB pathway signalling cascades. In an inactivated state, NF-κB is located in the cytosol as a protein complex with the inhibitory protein IκBα but TLR and NLR signalling leads to the phosphorylation of IκBα, its ubiquitination and degradation by the cell proteasome. Liberated NF-κB then translocates into the nucleus and induces the transcription of specific genes (Fig 1).

PRR signalling pathways play a key role in both the innate and adaptive immune responses, for example by influencing the skewing of naïve T cells, the regulation of regulatory T cells and activation of antigen presenting cells (APCs) such as dendritic cells (DCs) and macrophages. DCs are specialized APCs, that regulate both innate and adaptive immunity (recently reviewed [21]) and are found throughout the lamina propria of the intestine as well as in the gut-associated lymphoid tissues such as the Peyer’s patches. Most tissue resident DCs are immature and poorly immunogenic due to the low expression of MHC molecules and co-stimulatory molecules. However, contact with MAMPs or other danger signals induces PRR signalling and activation of the NF-κB pathway leading to maturation and activation. Mature DC express high levels of MHC, co-stimulatory molecules and cytokines required for antigen presentation and T cell activation, clonal expansion and differentiation [22]. The levels of cytokines produced by activated DCs are influenced by the nature of the TLR, CLR and NLR stimuli. This has important consequences for the induction of different T cell subsets and also our understanding of how different strains and species of lactobacilli can differentially modulate the immune response.

In intestinal epithelial cells (IEC), activated NF-κB can induce the production of a broad range of chemokines and cytokines including interleukins (ILs), tumour necrosis factors (TNFs), growth factors and inducible beta-defensins (BDs). These play a key role in maintaining barrier functions and homeostasis (reviewed in [23]). Endocrine, goblet and enterocytes of the intestinal epithelium express a range of PRR to sense the presence of microbes. In the human colon TLR2 and 4 were shown by immunohistochemical techniques to be expressed mainly in the enterocytes lining the crypts [24-26]. In the mouse, immunohistochemical analyses showed that TLR2, TLR4 and TLR5 are localized on both the follicle-associated epithelium (FAE) covering the Peyer’s Patches and the epithelium of the small intestinal villi and crypts [27] but TLR4 expression was relatively low. TLR2 is located only on the apical membrane of small intestinal IECs in the mouse and human [26,27]. In the intestinal epithelium excessive immune responses to non-pathogens are avoided by several mechanisms: (i) the regulation of TLR expression, (ii). TLR localization, (iii) differential apical and basolateral TLR signalling, (iv) negative feedback regulation of the NF-kB pathway and (v) the attenuation of NF-κB activation by commensal bacteria. These adaptations are part of several overlapping and intertwined mechanisms that maintain homeostasis of inflammatory innate responses in the gut.

### Recognition of lactobacilli via TLR2 signalling pathways

**Figure 2:**
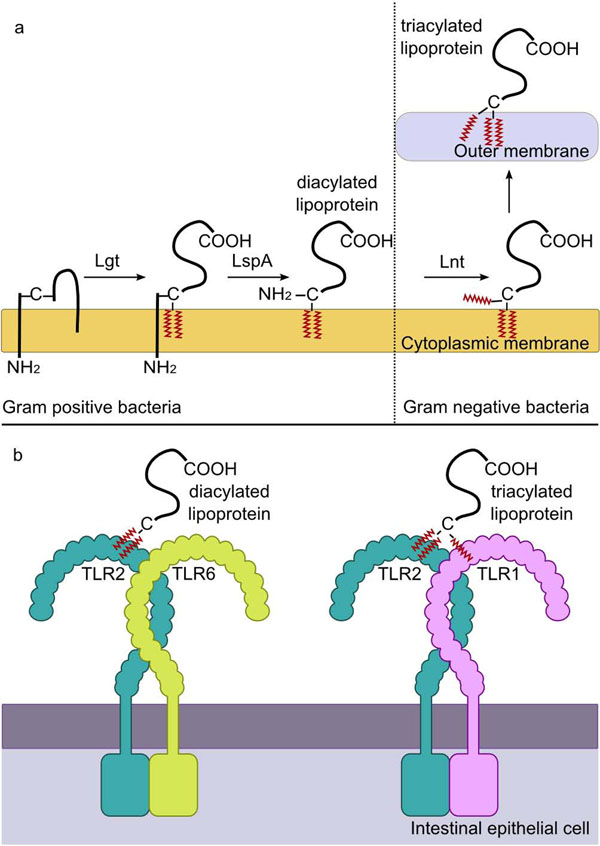
****TLR2 recognition of lipoproteins and LTA.**** Recognition of LTA and lipoproteins is mediated through binding of the lipid chains which anchor these molecules in the membrane. a). All lipoproteins possess a specific N-terminal lipoprotein signal which targets the protein for secretion and post-translational modification. In Gram-positive bacteria and Gram-negative bacteria the Lgt enzyme transfers a diacylglyceride group to the cysteine sulfhydryl group adjacent to the signal peptide cleavage site. Subsequently the signal peptide is cleaved just before the cysteine residue by LspA yielding a mature di-acylated lipoprotein. However, in Gram-negative bacteria and mycobacteria the Lnt enzyme attaches a third acyl group to the amino group of the N-terminal cysteine promoting its transport to the outer membrane. b). The diacyl lipid chains added by Lgt bind in a hydrophobic pocket in the extracellular domain of TLR2 and the head group of the peptide interacts with TLR6 to promote hetero-dimerization and signalling. In the case of tri-acylated lipoproteins the third lipid chain interacts with a hydrophobic channel in TLR1 to promote dimerization and signalling. This diagram is a modified version of Figure 1 published by Schenk et al., 2009 [101].

Cell wall components of the lactobacilli can potentially signal through binding to TLR2 in combination with TLR6. Over a decade ago bacterial lipoproteins were shown to recognize TLR2 [28,29]. Recognition is mediated through binding of the lipid chains which are post-translationally coupled to a specific N-terminal lipoprotein signal during secretion [30]. In Gram-positive bacteria and Gram-negative bacteria a prolipoprotein diacylglyceryltransferase (Lgt) transfers a diacylglyceride group to the cysteine sulfhydryl group adjacent to the signal peptide cleavage group (Fig 2). Subsequently the signal peptide is cleaved just before the cysteine residue resulting in the addition of two acyl (lipid) chains to the N-terminus of the lipoprotein. The acyl chains anchor the lipoprotein protein in the cytoplasmic membrane. However, in Gram-negative bacteria and mycobacteria which have an outer membrane an apolipoprotein N-acyltransferase (Lnt) enzyme attaches a third acyl group to the amino group of the N-terminal cysteine. This mediates transport of the lipoprotein to the outer membrane (Fig 2). Recent crystallographic structural data on binding of synthetic mimics of the lipoprotein membrane anchors to TLR1 and 2 or TLR2 and 6 have revealed that the diacyl lipid chains added by Lgt bind in a hydrophobic pocket in the extracellular domain of TLR2 (Fig 2). In the case of tri-acylated liopoproteins the third lipid chain interacts with a hydrophobic channel in TLR1 to promote dimerization and robust signalling [32]. In the case of TLR6 this hydrophobic channel is blocked preventing its binding to tri-acylated lipoproteins. Instead specific interactions with other parts of the di-acylated lipoprotein promote dimerization with TLR2 and signalling [31-33]. The co-receptors CD36, CD14 and lipopolysaccharide binding protein contribute to the recognition of LTA and TLR2-mediated signalling [34,35]. The anionic properties of the WTA and LTA may also contribute to immune signalling through their binding to scavenger receptors such as SRA which has been shown to bind purified LTA [36]. Apart from lipoproteins, TLR2 has been reported to bind the lipid chains anchoring lipoteichoic acid (LTA) in the cytoplasmic membrane of Gram-positive bacteria. In contrast the wall teichoic acids WTA are covalently attached to the peptidoglycan and presumably cannot signal through TLR2/6 as they lack a lipid anchor. LTAs and WTAs are anionic due to a D-ala substitution by the enzymes of the dlt operon. A Dlt- mutant of *Lactobacillus plantarum* strain NCIMB8826 was found to incorporate much less D-Ala in its TAs than the WT strain [37]. This defect significantly impacted on the immunomodulatory properties of the bacterium, as shown by a reduction in the secretion of proinflammatory cytokines by peripheral blood mononuclear cells and monocytes in co-culture with the *dlt* mutant and wild type strain [37]. This is also compatible with a study on a minimal synthetic structure of LTA showing that that the D-ala substitution of the polyglycerol backbone amplifies the response to the lipid anchor [38]. Furthermore, the amount of IL-10 produced in co-culture of PBMC with the *dlt* mutant was significantly increased compared to the parent strain [37]. These differences were shown to be TLR2 dependent implicating the interaction of LTA and D-ala substituted LTA with TLR2 in mediating the different immune responses. In a mouse model of colitis the Dlt- mutant was significantly more protective than its WT counterpart [37]. In contrast a *dltD* mutation in *L. rhamnosus* did not significantly alter cytokine production by intestinal epithelial cells and peripheral blood mononuclear cells in comparison to the wild type strain. Nevertheless the *L. rhamnosus dlt* mutant was more sensitive to anionic detergent, killing by to human beta-defensin-2 and showed altered cell shape, septation and increased rate of autolysis [39]. The reason for the differences between *L. plantarum* and *L. rhamnosus* are not clear but may be related to the species and strain-dependent differences in LTA and WTA composition. For example the cell walls of *L. rhamnosus* and *L. casei* appear to contain only LTA in contrast to other lactobacilli which also contain WTA [39].

Recently deletion of the phosphoglycerol transferase gene that primes the synthesis of bacterial LTA was reported in *L. acidophilus* strain NCK56 resulting in a strain lacking LTA [40]. Interestingly, the mutant strain NCK2025 down-regulated production of IL-12 and TNFα in co-culture with human monocyte derived dendritic cells but also significantly enhanced IL-10. The DC stimulated with the mutant and wild type strain also differed in their ability to induce proliferation of T cells isolated from MLN of mice which had been oral fed *L. acidophilus* for 4 consecutive days. T-cell proliferation was significantly abrogated in the LTA mutant-treated DCs co-cultured with T cells. Adding anti-IL10 antibody abrogated the suppression of T cell proliferation by the LTA mutant strain. Thus the T cell suppression was most likely due to the higher levels of IL-10 produced by DC cultured with the mutant strain than the wild type [40]. Furthermore, the NCK205 mutant was superior to the wild type strain in preventing dextran sulphate sodium (DSS) and colitis in the mouse CD4+CD45^RB high^ T cell transfer model. 	

### Recognition of lactobacilli genomic DNA via TLR9

Vertebrate innate immune system has evolved specific mechanisms to recognize bacterial DNA which differs from vertebrate DNA in respect of the frequency of methylated cytosin-guanodin dinucleotides (CpG motifs). In contrast to bacteria CpG motifs occur rarely in vertebrates and are mostly methylated [41]. TLR9 which is intracellular in immune cells but expressed on both the apical and basolateral recognizes bacterial CpG DNA and also synthetic unmethylated CpG oligonucleotide mimics (GpG-ODN) [42]. As the genome of *Lactobacillus* species differ in their C+G composition the TLR9 stimulatory capacity of different species is also likely to be different.

In polarized epithelial cells basolateral TLR9 stimulation with CpG-ODN was shown to activate the NF-κB pathway while apical stimulation prevented NF-κB activation conferring tolerance to chronic TLR challenges [43]. *In vitro* treatment of human epithelial cell monolayers with various bacterial DNA has been reported to have differential effects on IL-8 secretion [44]. DNA from pathogenic species of *Salmonella* but not from *Lactobacillius*, *Bifidobacterium* and *Streptococcus* species present in the VSL3 probiotic mixture induced IL-8 secretion in the human colonic cell line HT29. Furthermore, pre-treatment of HT29 cells with DNA from VSL3 delayed NF-κB activation, stabilized levels of IκB and attenuated IL-8 secretion in response to *Salmonella* DNA or TNFα [44]. Similar effects were also recently reported for HT29 and T84 polarized cell monolayers using purified DNA from *L. rhamnosus GG*[*45*].

CpG DNA and synthetic CpG-ODN stimulate macrophages, dendritic cells and monocytes to produce Th1 cytokines and NK cells to produce IFN-γ [46,47]. Prolonged (6-9 h) pre-exposure of macrophages to CpG DNA can however attenuate production of the proinflammatory cytokine TNFα following subsequent challenge with LPS via induction of IL-10 expression [48]. In contrast brief pre-exposure (1 – 3 h) of the RAW 264.7 mouse macrophage cell line to CpG-ODN augments the proinflammatory cytokine response to LPS [48]. Divergent effects of CpG-ODN have also been observed in experimental colitis where application of CpG-ODN in active disease exacerbated inflammation and prophylactic administration ameliorated colitis [49,50]. Recent studies in mice suggest that the prophylactic effects are due to the induction of regulatory properties in T cells [51].

VSL3 probiotic has been shown to reduce mucosal secretion of TNFα and IFN-γ and improve histologic disease in IL-10 deficient mice (Jijon 2004). This protective effect appears to be mediated by TLR9-triggered type I IFN as the addition of neutralizing antibodies against type I IFN abolished the anti-inflammatory effects induced by TLR9 agonists, whereas the administration of recombinant IFN-β mimicked the anti-inflammatory effects induced by TLR9 agonists [52]. The protective effect of VSL3 was also seen with non-viable or gamma-irradiated VSL3 probiotic, but not by heat-killed VSL3. Protection from colitis was seen with VSL3 in TLR2 and TL4 knockout mice but not TLR9 mice validating the role of TLR9 signaling in the probiotic mechanism [43].

In summary these studies suggest that unmethylated DNA fragments containing CpG motifs that are released from probiotics *in vivo* have potential to mediate anti-inflammatory effects via TLR9 signaling at the epithelial surface. Additionally, bacterial DNA from commensal microbiota or probiotic bacteria may contribute to the steady-state homeostasis via the enhancement of regulatory mechanisms.

### Immune signalling via peptidoglycan

Several studies report that preparations of macromolecular PGN (PGNpolymer) can activate NF-κB through human Toll-like receptors 2 (TLR2). However a recent study showed that purified peptidoglycan (PGN) isolated from *S. aureus* mutant lacking prolipoprotein diacylglyceryl transferase (Lgt) which couples the diacyl chains to lipoproteins in Gram-positive bacteria fails to stimulate TLR2 signalling [53]. This demonstrates that lipoproteins within the macromolecular structures of PGNpolymer, but not PGN itself, activate TLR2. Furthermore, an extensively purified monomeric PGN (PGNmonomer) failed to stimulate TLR2 signalling [53].

Peptidoglycan fragments are however recognized by the NOD1 and NOD2 members of the NLR family of PRR. There are more than 20 members of this receptor family in humans and they recognize a wide range of bacterial ligands and toxins as well as certain damage-associated molecular patterns (DAMPs) of the host cell [19,54]. NOD1 and NOD2 are the most well characterized NLRs and bind to the synthetic peptidoglycan mimics meso-diaminopimelic acid (DAP) and muramyl dipeptide (MDP), respectively [55,56] (Table 1). Both receptors are typically expressed in immune cells but in the intestinal epithelium only NOD1 is expressed throughout the epithelium. NOD2 expression is predominantly expressed in Paneth cells in the small intestine [57]. NOD1 and NOD2 activating ligands such muramyl dipeptide and Tri-peptide mesoDAP are transported into epithelial cells by the PepT1 di/tripeptide transporter which is highly expressed in the small intestine. In the colon PepT1 is poorly expressed but is induced during chronic inflammation [58]. NOD1 ligands have also been shown to enter cells through endocytosis, in a pH dependent manner [59]. NOD1 and NOD2 can also detect peptidoglycan fragments produced in the phagosome or phagolysosome of antigen presenting cells although the nature of the transporters involved in translocation to the cytoplasm remains unknown [60].

Typically the unmodified glycan strands of bacterial peptidoglycan consist of repeating units of β-1,4-linked N-acetylglucosamine and N-acetylmuramic acid but may undergo different enzymatic modifications such as O-acetylation [61]. In several lactobacilli the pentapeptide chains that are cross-linked between the D-alanine (D-ala) and L-lysine (D-lys) also differ from the consensus for lactic acid bacteria (L-ala, D-glu, meso-diaminopimelic acid or L-lys, D-ala, D-ala) [62]. For example in *L. casei* and *L. plantarum* the terminal D-ala is replaced by D-lactate. In addition the D-asparagine that forms the bridge between the pentapeptides, can be amidated in *L. acidophilus* and some other *Lactobacillus* spp. [62,63]. Highly O-acetylated peptidoglycan might be resistant to the hydrolytic activity of human lysozyme, thereby affecting the release of NLR stimulating PGN fragments and innate immune responses of antigen presenting cells such as dendritic cells and macrophages.

NOD2 alone does not activate dendritic cells but it has been shown to act a potent co-stimulator of the innate immune response exclusively in the presence of TLR signals [53]. Addition of highly purified PGNmonomer isolated from *S. aureus* in combination with TLR agonists up-regulated production of IL-12p70 and IL-23 in DC [53]. This synergistic effect was lost in dendritic cells from NOD2 deficient mice showing that NOD2 is the bone-fide receptor for highly purified PGN monomer.

### Influence of exopolysaccharides on immune signalling

In addition to PGN and teichoic acids, exopolysaccharides (EPSs) are also commonly associated with the cell wall of lactobacilli. The EPSs may be attached to the cell wall or secreted into the milieu. The structural diversity is large due to the presence of different sugar monomers and also variation in the modes of linkage, substitution and branching [64]. Some S-layer proteins of lactobacilli appear to be glycosylated [65] and other surface proteins attached to the cell wall may also be glycosylated [66]. EPSs and other cell wall polysaccharides could be recognized by one of several C-type lectin receptors (CLRs) that are involved in the recognition and capture of antigens by antigen presenting cells such as dendritic cells and macrophages.

The addition of heat-killed *Lactobacillus casei* Strain Shirota or its purified polysaccharide-peptidoglycan (PS-PG) complex decreased production of IL-6 in LPS-treated RAW 264.7 cells [67]. A role of the cell wall polysaccharide-1 (PS-1) was subsequently confirmed by Yasuda and colleagues who compared the immune-suppressive properties of PS-1 mutants with the wild type strain [68]. It is not known whether any lactobacilli express immunomodulatory zwitterionic polysaccharides like the prototype PSA found in a non-toxin producing *B. fragilis* strain. This PSA is degraded in the phagolysosome by oxidative depolymerisation to yield fragments that can be presented by the MHC class II molecule to CD4+ T cells carrying αβ-T cell receptors [69]. *B. fragilis* PSA has been shown to influence the special and temporal development of T cell responses during colonization of germ-free mice and it can protect against colitis by a mechanism involving the induction of regulatory T cells and IL-10 production [69].

Some bacterial pathogens such as *Mycobacterium tuberculosis* and viral pathogens such as HIV-1 escape immune surveillance by targeting the C-type lectin DC-SIGN (DC-specific intercellular adhesion molecule-grabbing non-integrin) on dendritic cells. Binding to this receptor is mediated by high mannose containing carbohydrate structures and affects maturation of DC and increases expression of IL-10. The result is reduced capacity of the DC to induce T cell-mediated responses against the pathogen. DC-SIGN binding activity has also been described for some strains of *Lactobacillus* spp. and is proposed to have an immunomodulatory effect [70,71].

### *In vitro* assays of immunomodulation by lactobacilli

*In vitro* co-culture assays with lactobacilli and different types of immune cells, such as human monocyte derived DCs, human PBMCs and mouse bone marrow derived DCs have often been used to assess the immunomodulatory potential of different species and strains (reviewed in [72]). IL-10 production is typically measured because it is an anti-inflammatory cytokine that suppresses IL-12 production and consequently IFN-γ production, thus favouring a T-helper 2 (Th2) or a regulatory T cell (Treg) response. In addition, IL-10 down-regulates antigen presentation and inhibits the activation of macrophages and thus the production of pro-inflammatory molecules and chemokines. IL-12 is also commonly measured as IL-12 production by DC is associated with the induction of T-helper 1 (Th1) responses. Furthermore IL-12 elicits IFN-γ production by T cells and by NK cells. There is a lack of evidence linking *in vitro* data to *in vivo* data for different strains but some studies indicate a correlation. Three studies have shown that the ability of different lactobacilli to induce a high ratio of IL-10/IL-12 or IL-10/TNF-α production in immune cells correlates with their capacity to provide significant protection in TNBS induced colitis in mice and rats [73-75]. Foligne et al., showed that the ranking of strains obtained on the basis of an *in vitro* IL-10/IL-12 cytokine induction ratio closely correlates the ranking of the *in vivo* ability of the strains to attenuate experimental colitis [73]. In contrast another study has shown that strains with similar cytokine ratios can have different outcomes in the mouse DSS model of colitis [76]. The reasons for their different immunomodulatory properties is not clear but may be due the levels of cytokines produced rather than the ratio and/or the survival and persistence of the strains *in vivo.*

Several studies have shown that the *in vitro* cytokine responses of human PBMC and DC to lactobacilli can be strikingly different depending on both the species and the strain [73,77-84]. As discussed further below different subsets of DC with distinct immunological phenotypes exist within the mucosal tissues so these *in vitro* models serve only as models to characterize the potential immunomodulatory properties of bacteria and or other factors (recently reviewed [72]). Despite these limitations there is evidence that the immune profile obtained in co-culture assays with bacteria and immune cells (especially for IL-10 and IL-12) can be predictive of their *in vivo* immunomodulatory activities [73,75,85].

**Figure 3:**
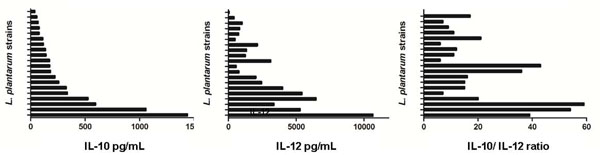
**IL-10, IL-12p70 production and the IL-10/IL-12p70 ratio by monocyte-derived dendritic cells stimulated with 20 different *L. plantarum* strains.** The graphs were produced using data from Meijerink et al., [69] based on one of the 5 representative donors. The different strains induce striking different amounts of these cytokines. Based on the IL-12 to IL-10 ratios it is clear that these cytokines can vary independently of each other resulting in strains with distinct pro-inflammatory and anti-inflammatory profiles.

Recently Meijerink et al., 2010 studied DC responses to 42 *L. plantarum* strains and the amounts of IL-10 induced cytokines ranged from 28 pg/mL to 1095 pg/mL (39 fold) for IL-10 and from 20-11996 pg/mL (600 fold) for IL-12 (Fig 3) [79]. From a comparison of IL-12 to IL-10 ratios it was clear that these cytokines can vary independently of each other resulting in strains with distinct pro-inflammatory and anti-inflammatory profiles. This remarkable diversity in the immunodulatory properties of different strains of the same species suggests that multiple factors can influence the phenotype in immune assays.

### Novel approaches to find immunomodulatory components of lactobacilli

Comparative genomic analysis is one approach to identify genetic loci linked to certain phenotypes and has been applied to *Lactobacillus* spp. to identify a genes involved persistence in the intestine [86] and mannose binding [71]. Similarly, the natural diversity in the immune response to different strains of *L. plantarum* has been exploited to identify novel genes influencing the dendritic cell response to *L. plantarum*[79]. In this study the secreted cytokine amounts or cytokine ratios for the *L. plantarum* strains were correlated with the presence or absence of specific genes by regression using the Random Forest algorithm. The genotypic data for each strain was based on comparative hybridisation to a whole genome microarray of strain WCFS1. This limited the analysis to genes present in WCFS1 but nevertheless several interesting loci were identified. The absence of several plantaricin-related genes correlated with higher IL-10 production in co-culture with DC. Six of these genes (*lp_0422*, *lp_0423*, *lp_0424a*, *lp_0424*, *lp_0425* and *lp_0429*) were present within the locus (*lp_0403* to *lp_0431*) required for plantaricin biosynthesis and secretion. Deletion mutants of the candidate genes were constructed in the WCFS1 strain in order to validate their anticipated effect on cytokine induction. All but one of the identified genes (a bile salt hydrolase) affected the immune response as predicted. All of the mutations introduced into the plataracin locus affected the immune response and were predicted to inhibit plantaracin production. Thus it is possible that the plantaracins directly affect the immune response of DC. Moreover, the expression of the *L. plantarum* plantaricin immunity protein PlnI is induced in the mouse intestine, suggesting that bacteriocins are produced *in vivo*[87].

Absence of a predicted transcriptional regulator gene (*lp-2991*) was also correlated with higher production of IL-10 and TNFα. A deletion mutant of *lp_2991*, led to a significantly higher secretion of IL-10, IL-12p70 and TNFα compared to the wild-type control. This gene lies upstream of *gtcA3*, a putative teichoic acid glycosylation protein. Upstream of *lp_2991* is a manganese transport gene *mntH2* which is orientated in the opposite direction. Transcriptome analysis and qPCR data supported the hypothesis that lp_2991 is a repressor of gtcA3 transcription and points to this enzyme as being a prime candidate for the altered immune response. Interestingly GtcA3 is predicted to glycosylate TAs including LTA which is a known TLR2 agonist capable of modulating immune cell responses. As pointed out above an LTA mutant and mutants affecting D-ala substitution in LTA have already been found to have effects on the immune response [37,40].

An additional approach to identifying new immunomodulatory molecules involves the screening of metagenomic clone libraries in high throughput cell-based screening assays for NF-kB modulation. The development of such an assay was recently reported for the functional screening of human gut metagenome libraries [88]. Recently, tools have become available for the automated analysis of high-content spatio-temporal cellular events using multi-colour fluorescence microscopy in a high-throughput fashion. Advances in software capabilities in the extraction of quantitative measurements from the acquired images and the management and interpretation of terabytescale image data allows the combinatorial analysis of multiple parameters such as fluorescence intensity, distribution and co-localization as well as changes in cell shape, size or movement in real time [89]. Such an approach has potential to identify new microbial molecules involved in binding and signalling with host-cells for example by the use of strain collections or random and gene-targeted libraries in specific *Lactobacillus* strains. Selected small inhibitor RNA (siRNA) knock-down libraries could also be used in high-throughput screens to identify host genes leading to specific responses to bacterial strains or molecules.

### Immunomodulatory mechanisms *in vivo*

**Figure 4:**
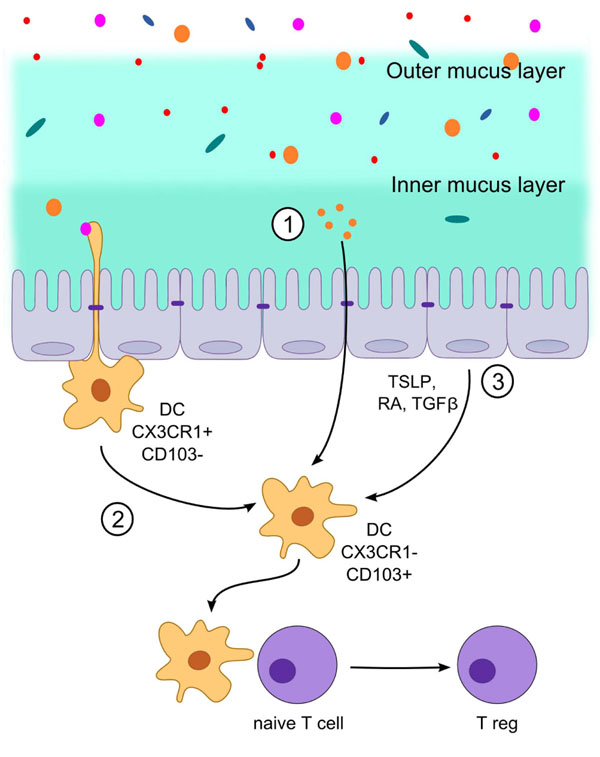
****Potential immunomodulatory mechanisms of probiotic lactobacilli**** (1). It is not yet clear precisely how oral administration of certain strains of probiotic lactobacilli can expand the Treg population in the mesenteric lymph nodes but it may involve direct activation of lamina propria (LP) CD103+ DC by MAMPs leading to up-regulation of MHC II and the co-stimulatory molecules needed for signaling and antigen presentation to naïve T cells in the lymphoid tissue. (2) Alternatively lactobacilli may be initially taken up by epithelial-associated CX3CR1^+^ DCs which could indirectly lead to maturation of the migratory CD103+ DC population. The oral administration of probiotic lactobacilli may also stimulate epithelial signaling and the production of cytokines such as TGFβ and TSLP as well as other factors which imprint a tolerogenic phenotype on resident CD103+ DC in the LP.

Several *in vivo* studies involving probiotic lactobacilli point to an immunomodulatory effect on intestinal DCs. Recently it was shown that administration of several strains of bacteria (including species of *Lactobacillus*, *Bifidobacterium* and *Streptococcus*) which had been selected *in vitro* to induce high IL-10 to IL-12 ratios were shown to expand the regulatory T cell population in the mesenteric lymph nodes [85]. Regulatory T cells (Tregs) are important in immunological tolerance due to the suppression of effector T cells at inflammatory sites and have therapeutic effects in models of inflammatory bowel disease, atopic dermatitis and rheumatoid arthritis. In the mucosa Tregs can be induced in the mucosal lymphoid tissues by a migratory population of tolerogenic CD103^+^ DCs distributed throughout the lamina propria (LP) of the whole intestine [90] (Fig 4). This population of DC is distinct from the epithelium-associated CX3CR1^+^ DCs and macrophages which participate in innate immune clearance and support inflammatory Th17 T cell responses [91,92]. The tolerogenic property of the CD103^+^ CX3CR1^-^ DC subset is determined by their local tissue environment and in particular by epithelial derived transforming-growth factor beta (TGF-β), retinoic acid (RA) and in humans, also thymic stromal lymphopoietin (TSLP) [93,94]. Interestingly, these factors are induced in IECs after apical interaction with bacteria *in vitro*. This suggests that that luminal contact with bacteria or bacterial factors can potentially modulate DC function in the LP. The CD103^+^ DC also express the enzyme indoleamine 2,3 dioxygenase (IDO) which is required for their tolerogenic function [95]. The immunosuppressive effects of IDO are linked to its catabolism of tryptophan and and/or production of immunomodulatory metabolites [95]. Most of our understanding of this tolerogenic mechanism is based on studies in the mouse but the migratory CD103^+^ DC subset is also found in the LP and mesenteric lymph nodes (MLNs) of humans suggesting the mechanisms are conserved.

It is not yet clear precisely how oral administration of lactobacilli can expand the Treg population in the mesenteric lymph nodes [85] but it may involve activation of LP DC leading to up-regulation of MHC II and the co-stimulatory molecules needed for signalling and antigen presentation to naïve T cells. This effect may be indirect e.g. by uptake of lactobacilli by the non-migratory DC subset associated with the epithelium (Fig 4). Another possibility is that oral administration of probiotic lactobacilli stimulates epithelial signalling and the production of cytokines such as TGFβ and TSLP as well as other factors which serve to imprint a tolerogenic phenotype on resident CD103^+^ DC in the LP (Fig 4).

A major gap in our knowledge about the mechanisms of immunomodulation by probiotics (including lactobacilli) concerns their fate *in vivo*. Studies on the thickness and physical state of the mucus layer throughout the intestinal tract has shown that it is thickest in the colon (~830 μm) and thinnest in the jejunum (~123 μm) [96]. If the mucus is removed by suction, a continuous, firmly adherent mucus layer remains attached to the epithelial surface in the colon (~116 μm) but in the small intestine this is much thinner (~20 μm) or absent [96]. The firm adherent layer of mucus in the colon is relatively devoid of bacteria [97] showing that the mucus layer is a significant physical barrier to contact of microbes with the epithelium. In the small intestine microbial contact with the epithelium may be more frequent due to the thinner layer of adherent mucus. Additionally the follicular epithelium covering the mucosal associated lymphoid tissue of the Peyer’s Patches (PP) in the small intestine is considered to be more accessible to antigens and bacteria present in the luminal compartment.

Evidence that the mucosal tissue responds to orally consumed lactobacilli comes from recent studies on the mucosal transcriptome responses to orally consumed *L. plantarum* in humans [98,99]. In the study of Baarlen et al., [99] 3 preparations of *L. plantarum* were tested in a double-blind placebo-controlled cross-over design: (i) the logarithmic-phase of growth, (ii) the stationary-phase of growth, and (iii) heat-killed ‘‘stationary’’ bacteria. The microarray expression profiles of duodenal biopsies taken 6 h after consumption displayed striking differences including genes encoding NF-kB subunits and inhibitors of NF-kB signalling pathways. In a follow study transcriptomes of proximal small intestine biopsies from healthy volunteers were obtained after consumption of three widely used probiotic strains of different *Lactobacillus* species (about 1 x 10^10^ bacteria) and a placebo control according to a randomized double-blind cross-over study design [100]. At 6 h each *Lactobacillus* strain induced markedly different expression profiles in the human mucosa, supporting other evidence from *in vitro* and *in vivo* studies that the beneficial properties of probiotics are strain dependent. Notably, there was a large person-to-person variation in the transcriptome response and a high co-efficient of variation for some of the chemokine and cytokine effectors which could help to explain the outcomes of probiotics reported in human studies.

### Influence of lactobacilli on antibody responses and immunity

Lactobacilli have been shown to increase the immunogenicity of orally administered vaccines such as rotavirus [101], polio [102], cholera [103] and influenza virus vaccine [104]. In the study on cholera vaccination several probiotic strains were tested for their effect on antibody responses to the commercial oral cholera vaccine (Dukoral, SBL Vaccin AB, Stockholm, Sweden). The probiotic strains were given daily over a 3 week including the period in which the oral vaccine was administered. Immunoglobulin serum concentrations tended to increase in most probiotic treated groups and between day 0 and day 21 significant increases in IgG were observed for *Bifidobacterium lactis* B1-04 and *Lactobacillus acidophilus* La-14 compared to controls (P= 0.01). However, no effect was observed on the final end point serum antibody titres to the vaccine or the specific IgA concentration in saliva [103]. Consumption of LGG or *L. acidophilus* CRL431 for five weeks doubled neutralization titres to an oral polio vaccine given in the fifth week [102]. In the group consuming probiotics marked increases in polio-virus specific IgA was also observed after vaccination. The adjuvant effect of LGG was also evaluated in conjunction with D x RRV rhesus-human reassortant live oral rotavirus vaccine in 2-5-month-old infants. Infants who received LGG showed an increased response with regard to rotavirus-specific IgM secreting cells on day 8 after vaccination. In infants receiving LGG rotavirus IgM seroconversion was not significantly different to placebo. However, rotavirus IgA seroconversion was detected in 26/28 (93%) of cases in the LGG group versus 20/27 (74%) infants given the placebo (p = 0.05) suggesting that LGG has an immunostimulating effect on oral rotavirus vaccination. Oral consumption of non-viable *Lactobacillus pentosus* strain b240 enhanced secretory IgA concentrations in the saliva of healthy volunteers [105] and more recently this strain was shown to increase survival time of mice infected with a lethal dose of influenza virus [104]. The adjuvant and immunomodulatory effects of lactobacilli on immunity are also evident from many studies utilising lactic acid bacteria as vaccine delivery vehicles for mucosal immunization [23,106].

There is also evidence that orally consumed probiotics can stimulate immune responses to respiratory pathogens [107]. Furthermore a recent study on orally consumed LGG as an immune adjuvant for live attenuated influenza vaccination in healthy adults which showed significantly improved protective serum responses to one of the three viruses present in the vaccine [108].

### Innate signalling and barrier function

Epithelial cells play a key role in directing the responses in the gut and maintaining homeostasis via the sensing of microbes or MAMPs (reviewed in [23]). Commensal bacteria are not ignored but dynamically controlled via many complex overlapping and intertwined mechanisms involving IECs and signals from the microbiota. Inflammation is avoided through the differential signalling and expression of receptors on the apical and basal membranes of the epithelium and via the regulation of pattern-recognition receptor expression and activity [17,18,43]. Moreover TLR signalling has been shown to have a protective role in the intestine [109,110]. Several lactobacilli have been reported to enhance barrier function and/or protect against barrier disruption by pathogens *in vitro* (reviewed recently [111,112]). Different strains of *Lactobacillus plantarum* have been reported to prevent the reduction in trans-epithelial resistance across epithelial monolayers caused by pathogenic *E.coli* when co-cultured with the pathogen. This could be due to inhibition of the pathogen or enhancement of the barrier function by modulating TJ composition [113-115]. Lactobacilli have also been reported to reverse the barrier dysfunction caused by proinflammatory cytokines *in vitro*[116].

In animal models *L. plantarum* strains have been shown to reduce the hyper-permeability associated with experimental enterocolitis [117] and biliary obstruction [118]. Recently a study was performed in human volunteers which showed that perfusion of *L. plantarum* into the duodenum increased the localization (immunofluorescent staining) of occludin and ZO-1 in the epithelial TJs of tissue biopsies [119]. TJ modification was mediated by TLR2 ligands and conferred protection against disruption of the TJs by phorbol ester *in vitro*. These results are in agreement with recent studies in mice showing protection from colitis by administration of TLR2 ligands [109]. The precise signalling mechanisms leading to TJ modulation by lactobacilli are not clear but specific PKC isoforms have been implicated from *in vitro* studies with TLR2 agonists [109]. This does not exclude the involvement of other mechanisms and receptors in TJ modification by lactobacilli such as EGF, the ERK1/2 members of the MAPK family [120-122].

## Conclusions

Lactobacilli can elicit innate and adaptive immune responses in the host is via binding to pattern recognition receptors (PRR) expressed on immune cells and many other tissues including the intestinal epithelium. The di-acylated membrane anchors of lipoproteins and lipoteichoic acids bind to TLR2 and TLR6 promoting dimerization and MyD88-mediated activation of the canonical pathway of NF-kB. The capacity of different species to stimulate TLR2 signalling varies considerably, even at the strain level for those species which have been extensively tested (unpublished Taverne, Meijerink and Wells). This may reflect differences in the expression level of certain lipoproteins, their release into the medium, the amount of LTA and its structure/ modification and the effect of shielding factors such as exopolysacchrides. Strain-dependent differences in MAMPs such as LTA, the peptidoglycan structure and non-methylated CpG motifs could explain why the *in vitro* cytokine responses of human PBMC and DC to lactobacilli can be strikingly different depending on both the species and the strain. Furthermore, sugars present in the cell wall and the post-translational modification of proteins with carbohydrates can also modulate the immune response by binding to C-type lectin binding receptors. It seems likely that more new strain-specific genes and factors that modulate innate recognition by the host will be discovered using gene-trait matching, functional metagenomic screens, and high throughput automated microscopy [79,88].

*In vivo* lactobacilli have been successfully used to modulate inflammatory diseases, enhance barrier functions and stimulate immunity. Evidence linking these beneficial properties to *in vitro* immune data is sparse due to the lack of comparative studies with different strains of probiotics *in vitro* and *in vivo*. Nevertheless, the immune profile obtained in co-culture assays with bacteria and immune cells (especially for IL-10 and IL-12) has been shown to be predictive of their anti-inflammatory properties [73,85]. Studies in mice indicate that certain strains of probiotics can be used prevent or treat inflammatory diseases via a mechanism which increases mucosal Treg cells [85]. This most likely involves the mucosal CD103+ DC that traffic between the lamina propria and draining lymphoid tissue in humans and mice. The tolerogenic phenotype of these DC is imprinted by secreted factors produced by epithelial cells and other cells and thus may be subject to regulation by epithelial interactions with the luminal microbiota. Increasing evidence also suggests that induction of epithelial signalling by non-pathogenic commensals in the lumen can modulate barrier functions and regulate inflammatory signalling [Karczewski 2010; Neish 2000; Kelly 2004}. Other probiotic mechanisms are likely to include modulation of the T effector subsets and the CX3CR1+ LP cells that can sample bacteria through the epithelium [93]. A major challenge for the future will be to gain a better understanding of how probiotics can modulate DC function in the LP and DC *in vivo* especially in humans where sampling of immune cells at mucosal sites is difficult to the invasive nature of the technique.

## Competing interests

The author has no competing interests.
